# Water Treatment for Atrophy

**Published:** 1897-07

**Authors:** 


### "WATER TREATMENT FOR ATROPHY."

"Mr.——, who is being treated at —— Hospital for atrophy, will stay in a bath-tub filled with water sixteen hours a day. This makes the sixth day of such immersion. He has shown such wonderful improvement that the treatment may become generally adopted. It will be named the —— treatment, in honor of the doctor who first tried it. Dr. ——, in explanation of his experiment, said he was struck by the fact that vegetables and fruits will become revived and fresh by immersion in water, and he could not see why humans should not thrive under such treatment."

We had long been familiar with the action of water on dried apples, but never had it occurred to our obtuse minds that the same treatment could be applied in cases of atrophy. We are sure that the profession will welcome the great discovery of Dr. —— with joy, that due credit will be accorded him for the same and that his name in the future will be classed with the great men in the profession who have added so much to the advancement of medicine during the latter half of the nineteenth century.

The above clipping from one of our metropolitan daily newspapers, is a fair illustration of a form of medical advertising that is seen almost every day, a form of advertising that is in itself unique, inexpensive, deplorable, and to the honest physician disgusting; a form of advertising, that, when compared with the advertisement of the ignorant quack makes the latter appear as a man actuated by noble motives. We are treated sometimes with the description of a surgical operation, often illustrated, that is to work a great innovation in surgery, again it is some new remedy that is to cure some incurable disease.

It will be noted that in all these advertisements the patient has just been made to undergo the operation if it be a surgical case, or is passing through the treatment if it be a medical case; we never hear of the final results in the given case, that is wholly unnecessary; if the second chapter were published, we fear frequently the advertisement would not prove a drawing card for the doctor.

The reporter of the daily paper is sagacious, acute and ever ready to publish anything of a sensational nature, but he could get hold of these cases only by or through the consent of the attending physician. It is time the profession sat upon this class of medical advertising, and gave the physician that allows himself to be interviewed with this object in view to understand that such conduct on his part is, to say the least, unprofessional.—A. B. B. in *Atlantic Medical Weekly*.

